# Advances in green-synthesized magnetic nanoparticles for targeted cancer therapy: mechanisms, applications, and future perspectives

**DOI:** 10.1186/s40001-025-03804-9

**Published:** 2026-02-07

**Authors:** Ahmed M. El-khawaga, Omneya Ibrahim Youssef, Omnia A. El-dydamoni, Waleed Mahmoud Ragab, Rehab Abd Elfattah Mohammed

**Affiliations:** 1 Department of Basic Medical Sciences, Faculty of Medicine, Galala University, New Galala City, Suez, 43511 Egypt; 2 Ibn Sina National College of Medical Sciences, Jeddah, Saudi Arabia; 3 Medical Microbiology and Immunology, Faculty of Medicine, Al-Azhar University for Girls, Cairo, Egypt; 4 Anatomy and Embryology Department, Faculty of Medicine, Galala University, Galala, Suez, 43511 Egypt; 5 Department of Internal Medicine, Ibn Sina National College of Medical Sciences, Jeddah, Saudi Arabia

**Keywords:** Magnetic nanoparticles, Cancer therapy, Drug delivery, Treatment

## Abstract

Magnetic nanoparticles (MNP) have gained significant attention for their potential in cancer therapy, particularly in targeted drug delivery, imaging, and hyperthermia treatments due to their unique magnetic properties, biological compatibility and applicability. This literature review focuses on recent progress in the green-synthesized MNP, explores their mechanisms of drug delivery, and critically evaluates their clinical applicability. The gaps in the literature that this review addresses include the inconsistency in nanoparticle size and surface properties, the limitations in achieving sustained and predictable drug release, and the difficulties in maintaining long-term stability in physiological conditions. It also discusses potential future development, including smart nanotechnology, individual medicine, and AI-acquired platforms. These findings show how MNPs can increase precise oncology by increasing medical effect, reducing toxicity and lightweight real-time monitoring of treatments.

## Introduction

Cancer is one of the leading causes of death globally, accounting for millions of lives lost each year [1]. Efforts to develop more precise and effective cancer therapies have brought nanotechnology to the forefront as a paradigm shift in cancer care [2]. Nanotechnology is a concept that integrates the design and application of materials at the nanometer scale, allowing us to manipulate biological processes at the cellular and molecular level. Although there are many different types of nanoparticles that have been studied, magnetic nanoparticles (MNPs) are of particular interest because of their unique magnetic properties, surface modifiability, and the ability to interact with external magnetic fields [3]. These factors also make MNPs suitable for many applications, such as targeted drug delivery, imaging, diagnostics, and hyperthermia treatments [4]. However, traditional MNP synthesis methods, including chemical and physical approaches, often involve hazardous chemicals, high energy consumption, and environmental concerns. These methods also pose challenges related to the reproducibility of nanoparticle size, surface properties, and scalability, which are critical for achieving consistent therapeutic outcomes [5]. In contrast, one of the most exciting developments in the field is the emergence of the green-synthesized magnetic nanoparticles [6, 7]. Green synthesis utilizes biological agents, such as plant extracts, microorganisms, and natural enzymes that can produce nanoparticles over mild, environmentally friendly conditions [8]. This decreases the environmental burden for nanoparticle production and improves biocompatibility making them more suitable than synthesis processes [6, 9]. Green-synthesized MNPs have many advantages for drug delivery systems. Because of their small size, they can penetrate tissues deeply allowing for accumulation to the tumor site by enhanced permeability and retention effect (EPR) [10]. Their magnetic core provides an external field-guided navigation which can facilitate selective targeting of the tumor and delivery of chemotherapeutic agents directly to the tumor area while limiting exposure to the body which minimizes side effects [11]. Drug delivery is also possible by functionalization of the surface of the nanoparticle with ligands, antibodies, or polymers allowing active targeting and controlled drug release [12]. The purpose of this literature review is to investigate the use of green-synthesized magnetic nanoparticles for cancer therapy. The literature will begin with limitations of cancer treatment modalities and establishing how drug delivery systems can circumvent limitations of cancer therapies. It will discuss synthesis, structure, and functional properties of MNPs and the advantages of green synthesis method. The literature will cover mechanisms of targeted delivery of drug and provide examples/case studies and experimental support of green-synthesized MNPs effect on efficacy in both in vitro and in vivo. Additionally, there will be discussion of potential issues with stability of nanoparticles, scalability of synthesis methods, regulatory approval, and long-term safety. Finally, the literature will arrive at a conclusion of future thoughts with advances in nanoparticle design, potential for greater synergy with combination therapy, and desire to contribute to advancing personalized medicine. Overall, reflections on current research with consideration of gaps in knowledge demonstrate further dialog about the future of nanotechnology in cancer treatment.

## Overview of cancer therapy challenges

Traditional cancer treatments (chemotherapy, radiation, and surgeries) have been foundational approaches for cancer treatment for decades [13]. Although these approaches have improved survival rates in some cancers, they do have substantial disadvantages. One major concern in current cancer therapies is lack of selectivity, and hence, healthy cells are included as targets alongside malignant cells [14]. Nonselective activity leads to side effects, including fatigue, nausea, immunosuppression, alopecia, and opportunistic infections. Such side effects not only reduce the patient’s quality of life but may also limit the dose and duration of treatment, compromising its overall effectiveness [15]. Chemotherapy, in particular, is known for its systemic toxicity. Although chemotherapeutic agents are designed to target rapidly dividing cells, many normal cells such as those in the gastrointestinal tract, bone marrow, and hair follicles also divide rapidly and become unintended targets [16]. Furthermore, the pharmacokinetics and pharmacodynamics of chemotherapeutic agents can vary widely between patients, leading to inconsistent therapeutic responses. Resistance to chemotherapy, both intrinsic and acquired, is another major hurdle [17]. Tumor cells may develop mechanisms to pump out drugs (efflux), repair DNA damage, or activate survival pathways, making them less responsive to treatment [18]. Radiation therapy, though localized, poses challenges of its own. High-energy radiation can damage surrounding healthy tissues and cause long-term side effects, such as fibrosis, secondary malignancies, and organ dysfunction. Moreover, some tumors are radioresistant and may not respond adequately even at high radiation doses, increasing the risk of recurrence [19]. Surgical intervention, while effective in removing localized tumors, is limited by tumor accessibility and the risk of incomplete resection. In cases of metastatic disease, surgery is often not viable, and recurrence is common due to residual cancer cells or micrometastases that evade detection [20]. Tumor heterogeneity—both within a single tumor (intra-tumoral) and between patients (inter-patient)—affects how cancers respond to therapy [21]. Some cells may be more resistant to drugs due to genetic mutations, varying levels of oxygen (hypoxia), or differences in the tumor microenvironment [22]. Hypoxic regions within tumors, for example, are less susceptible to radiation and certain chemotherapies, leading to incomplete eradication and eventual relapse [22].Hydrophobic medicines often require high doses or toxic solvents to achieve medical concentrations, increasing systemic poisoning. In addition, biological barriers such as blood–brain barriers limit the effectiveness of systemic agents for brain tumors or metastases [23]. Multidrug resistance (MDR) constitutes another malignant component of having successfully treated malignancies [24]. MDR is, most frequently, meditatively limited by potent pumps such as P-glycoprotein, which actively extrude therapeutic drugs from cancer cells to lower intracellular levels to below the effective concentration [24]. Other mechanisms of drug resistance may include increased DNA-repair abilities, modified drug targets, and evasion of apoptosis [18]. New therapeutic modalities are needed to address these complex issues. These new modalities include precision medicine, targeted therapies, immunotherapy, and nanotechnology drug delivery modalities [25]. This advancement can improve the therapeutic index by lowering drug specificity, decreasing side effects, and increasing the therapeutic index by avoiding the drug resistance mechanisms.

## Role of drug delivery systems in cancer treatment

The drug delivery system (DDS) is an essential component of Modern Cancer Medicine, which is meant to provide new and safe techniques of enhancing cancer treatment effectiveness [26]. Traditional chemotherapy is burdened with multiple issues, such as poor targeting ability, and rapid systemic clearance, and dose-limiting toxicity [27]. DDS have been specifically established to tackle these issues, as it utilizes these issues as means of targeting tumor sites, which provides efficiency, safety, and site-specific drug delivery [28]. It brings about high local drug concentrations, extended drug retention on tumors, and minimal off-target side effects. While most known DDS platforms, liposomes and polymers, have been widely studied, MNP has specific characteristic that traditional systems do not offer [29]. In most cases, MNP particles with iron-oxide core can be anchored to specific sites with outside magnetic field. This magnetic targeting allows for specific targeting of therapeutic agents, minimizes systemic dissemination, and increases treatment efficacy [30]. Another essential attribute of MNP-DDS is its multipurpose. Aside from drug delivery, MNP can also be used for clinic imaging such magnetic resonance imaging (MRI) and magnetic hyperthermia. This double role of therapy and diagnosis is known as 'Therrnostics', which is a rapidly growing area in personal medicine [30]. Controlled drug release is another important component of DDS. With MNP, it can be achieved by optimizing temperature, pH or enzyme-sensitive coating that releases the substance in response to specific stimuli found in tumor microelements. This ensures that the substance remains preserved during circulation and is released only when it reaches cancerous tissue [31]. MNP can be loaded with both chemotherapy and sensitizing molecules, which provides the possibility of complex effects. In addition, surface modification enables ligand, antibodies, or aptamer active targeting of specific cancer cell markers, which further improves selectivity [32]. Studies have demonstrated that MNPs can significantly improve the accumulation of therapeutic agents within tumors, thereby increasing the local concentration of drugs while minimizing side effects in healthy tissues [33, 34]. Moreover, the surface functionalization of MNPs with targeting ligands, such as antibodies or peptides, has been shown to further enhance this effect, ensuring that drugs are delivered specifically to cancer cells [35, 36].

Drug delivery systems (DDS), including magnetic nanoparticles (MNPs), possess inherent limitations that diminish their efficacy as therapeutic options. Challenges include unpredictable pharmacokinetics, rapid elimination, and nonspecific distribution [37]. Nanoparticles exhibit diverse dimensions, possess distinct surface characteristics, and deliver pharmaceuticals at various rates. This complicates the attainment of consistent outcomes and hinders the replication and regulation of drug release [38]. Concerns also exist regarding biocompatibility, toxicity, and systemic adverse effects that may arise due to immunological responses and coagulation [39]. Magnetic targeting has obstacles related to insufficient tissue penetration and difficulty in accurately locating the desired site. Another significant problem that must be addressed before DDS can be extensively utilized in medicine is the development of a cost-effective and manufacturable solution [40].

## Green synthesis of magnetic nanoparticles

**Fig. 1:**
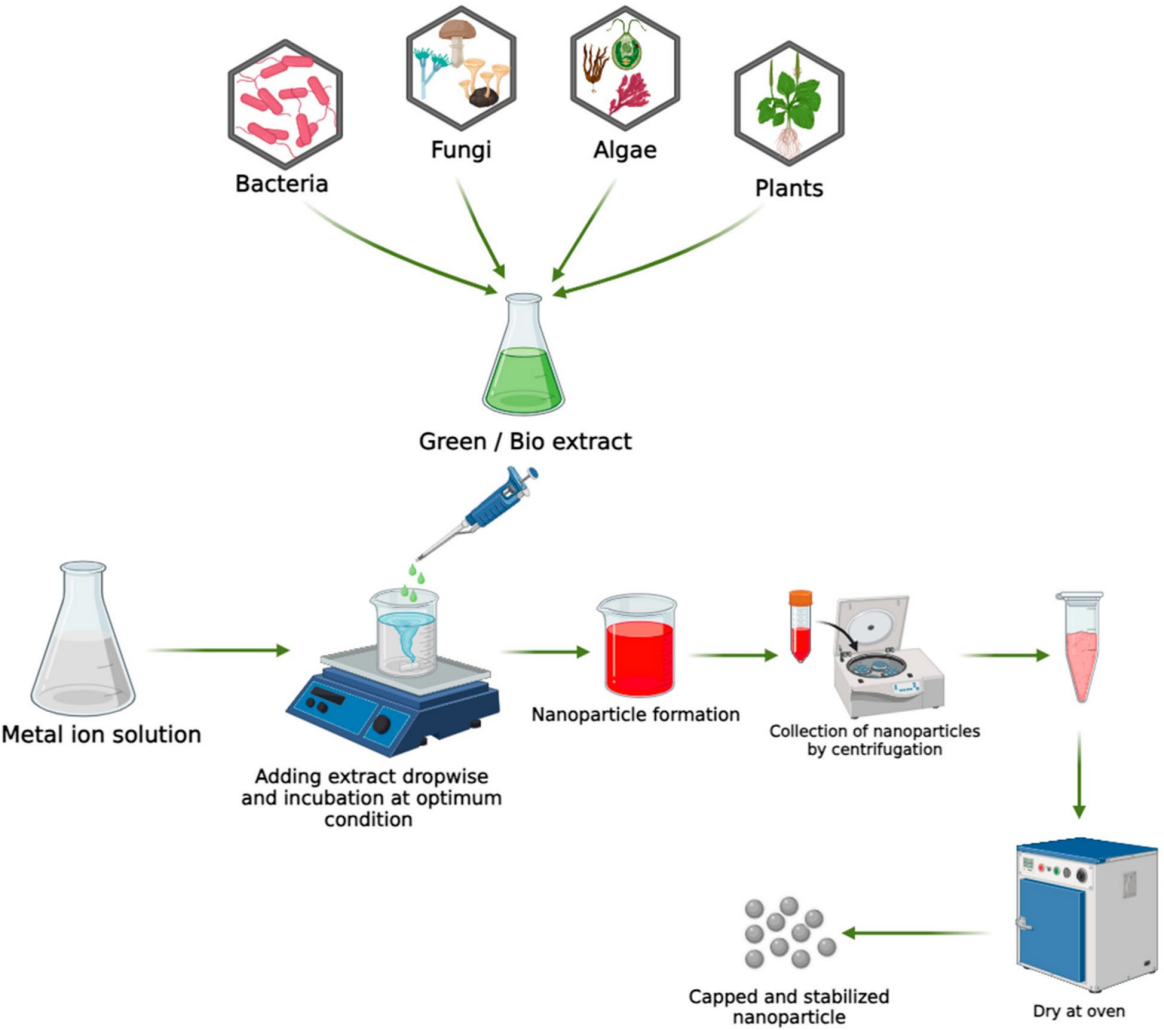
The schematic diagram shows the biosynthesis of NPs [51]

Green synthesis of MNPs has proven to be a permanent and environmentally friendly approach in nanotechnology. It benefits from naturally derived materials and biological processes to produce nanoparticles with toxicity and increased biocompatibility [41]. Traditional chemical and physical methods often require high temperatures, dangerous reagents, and detailed layouts, which can be energy-intensive and environmentally harmful [42]. Contrary to this, green tanks appoint synthesis extracts, bacteria, fungi, and other biological systems to facilitate the formation of NPs under mild conditions [43]. Plant mediation synthesis is particularly attractive due to its simplicity and abundance of phytochemicals, such as polyphenols, flavonoids, terpenoids, alkaloids, and tannins [44]. These compounds act as both reduced and stabilizers, which convert the metal salts such as iron chloride into magnetite (Fe_3_O_4_) or maghemite (γ-Fe_2_O_3_) NPs. The benefits include low costs, ambient temperature operations, and minimum processing after hypothesis [45]. In addition, the plant-based synthesis methods are easily scalable and compatible with industrial needs [46]. Microbial synthesis is another green passage that uses bacteria, fungi, and algae to convey the production of MNP [47]. Microorganisms have enzymatic systems and metabolic passages that enable them to reduce metal ions and control the particle size and size. For example, the *Bacillus subtilis* and *Aspergillus niger* have been used with success to biosynthesized iron-oxide NPs [48, 49]. These NPs often show natural coatings or surface modifications that increase the spread in the aquatic environment and facilitate interaction with cells. As a result, green-synthesized MNPs are quickly assessed for applications in drug distribution, diagnosis, imaging, and hyperthermia [50]. Figure 1 shows the concept of green synthesis, which highlights the role of plant extracts and microbial agents in converting metal ions to NPs.

Bioactive compounds not only reduce ions but also cap particles to prevent agglomeration and improve stability. It is consistent with the principles of green chemistry and provides benefits in terms of safety, costs, and purposes in biomedical regions [52]. The continuous detection of biological agents and their mechanisms will increase the accuracy and scalability of this promising synthesis approach. Phytochemicals, such as polyphenols and flavonoids, convert Fe^3^⁺ ions into iron-oxide nanoparticles through green synthesis. The reducing agents also help limit the movement of the particles. The nucleation process is influenced by temperature, pH, and phytochemical content. These factors dictate the size and shape of the particles. These natural materials regulate nanoparticle growth, resulting in more uniform morphologies. The parameters governing particle formation influence their crystallinity and magnetic properties. Enhanced crystallinity typically correlates with superior magnetic performance, facilitating the nanoparticles' responsiveness to external fields, so ensuring precise drug delivery.

Despite the environmental advantages of green synthesis methods, the production of reliable and reproducible MNPs remains challenging [53]. Diverse biological factors, such as plant extracts or microbes, can influence the size, charge, and stability of particles. These discrepancies can alter the drug's release and efficacy, so it is crucial to standardize green synthesis methodologies for medicinal applications [54].

## The main characteristics of magnetic nanoparticles

Magnetic NPs has emerged as one of the most promising nanomaterials in biomedicine due to its unique physical and chemical properties. With iron-oxide-based materials, we typically expect to be able to synthesize materials like iron oxides magnetite (Fe_3_O_4_) or maghemite (γ-Fe_2_O_3_) [55]. Due to the superparamagnetic properties of these types of materials, they will respond to an external magnetic field (i.e., show magnetism) but will not possess any magnetism once removed. These properties of superparamagnetic nanoparticles are advantageous for medical applications such as temporary magnetic targeting which can be advantageous in both therapeutic administration and in magnetic resonance imaging (MRI) detection [56]. With MNPs, the surface-to-volume ratio will increase also increasing its reactivity, allowing for easy surface behavior and increasing the number of functional groups on the surface making MNP settling a versatile platform for biomedical applications [56]. MNPs can also be modified through surface decoration to allow for long circulation time, bioavailability, and targeted interaction to specific cells/tissues [57].

MNPs also exhibit favorable optical and electrical properties, which can be harnessed for biosensing and thermal ablation therapies [58]. Their stability in biological environments is improved through coatings, such as polyethylene glycol (PEG), dextran, or silica, which prevent aggregation and improve dispersion. These coatings further help in reducing immunogenic responses [59].

**Fig. 2:**
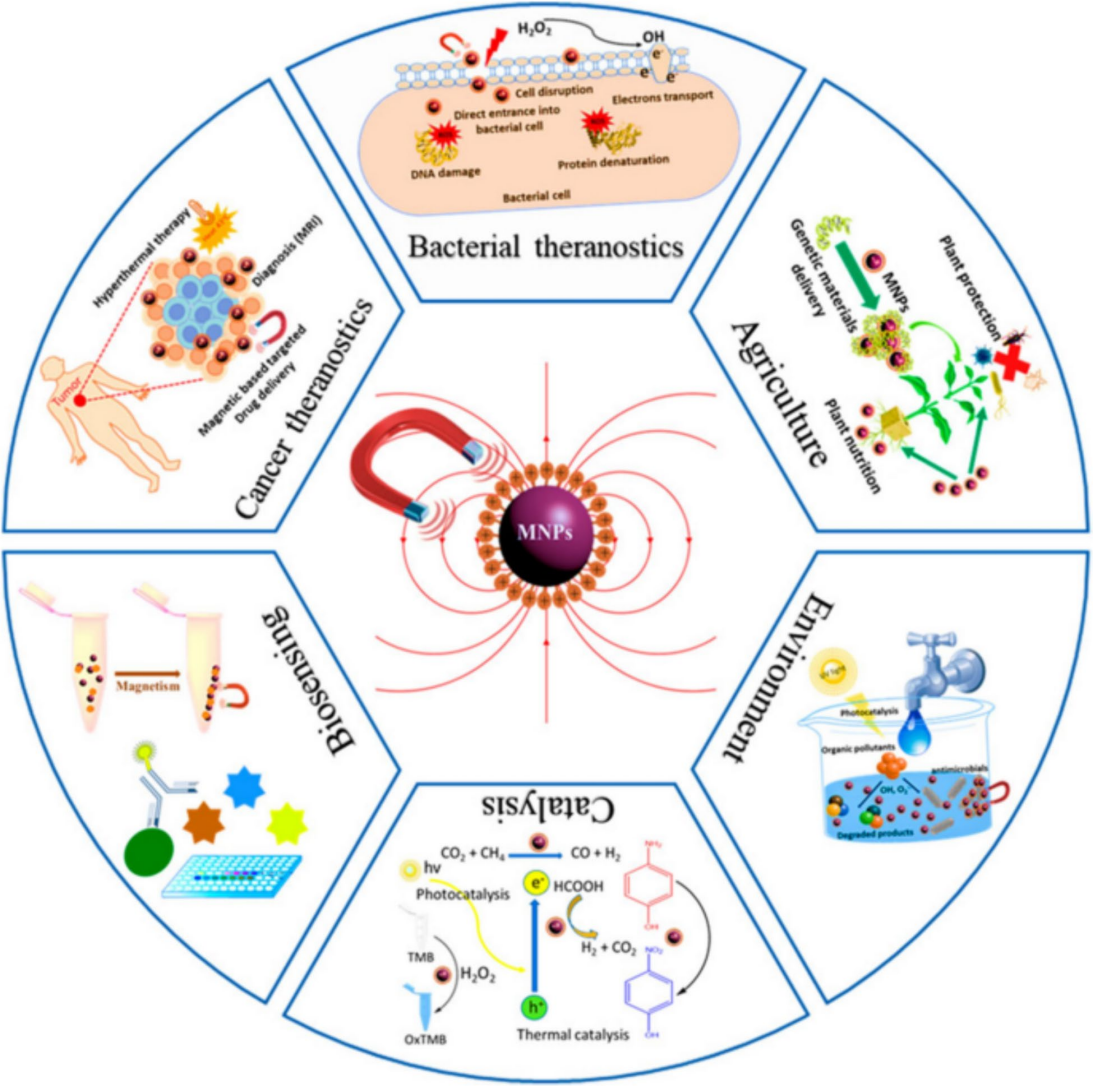
Biomedical applications of magnetic nanoparticles. Adapted from [61]

MNPs are ideal candidates for a range of biomedical applications due to their superparamagnetic nature, customizable surfaces, and excellent biocompatibility. Figure 2 shows that magnetic nanoparticles (MNPs) can be used in many different biomedical ways, such as for drug delivery, imaging, and hyperthermia treatment. The applications shown show how MNPs can be used for targeted drug delivery and real-time monitoring using imaging techniques like magnetic resonance imaging (MRI), because they are superparamagnetic. The figure backs up the claim that MNPs are a versatile way to treat cancer, because they can be used for both diagnosis and treatment in one system [60].

### Structural and magnetic properties

Structural and magnetic properties of MNPs play an important role in determining their performance in biomedical applications [62]. These properties are affected by many factors, including particle size, shape, crystallinity, and types of iron oxides used [63].

**Fig. 3:**
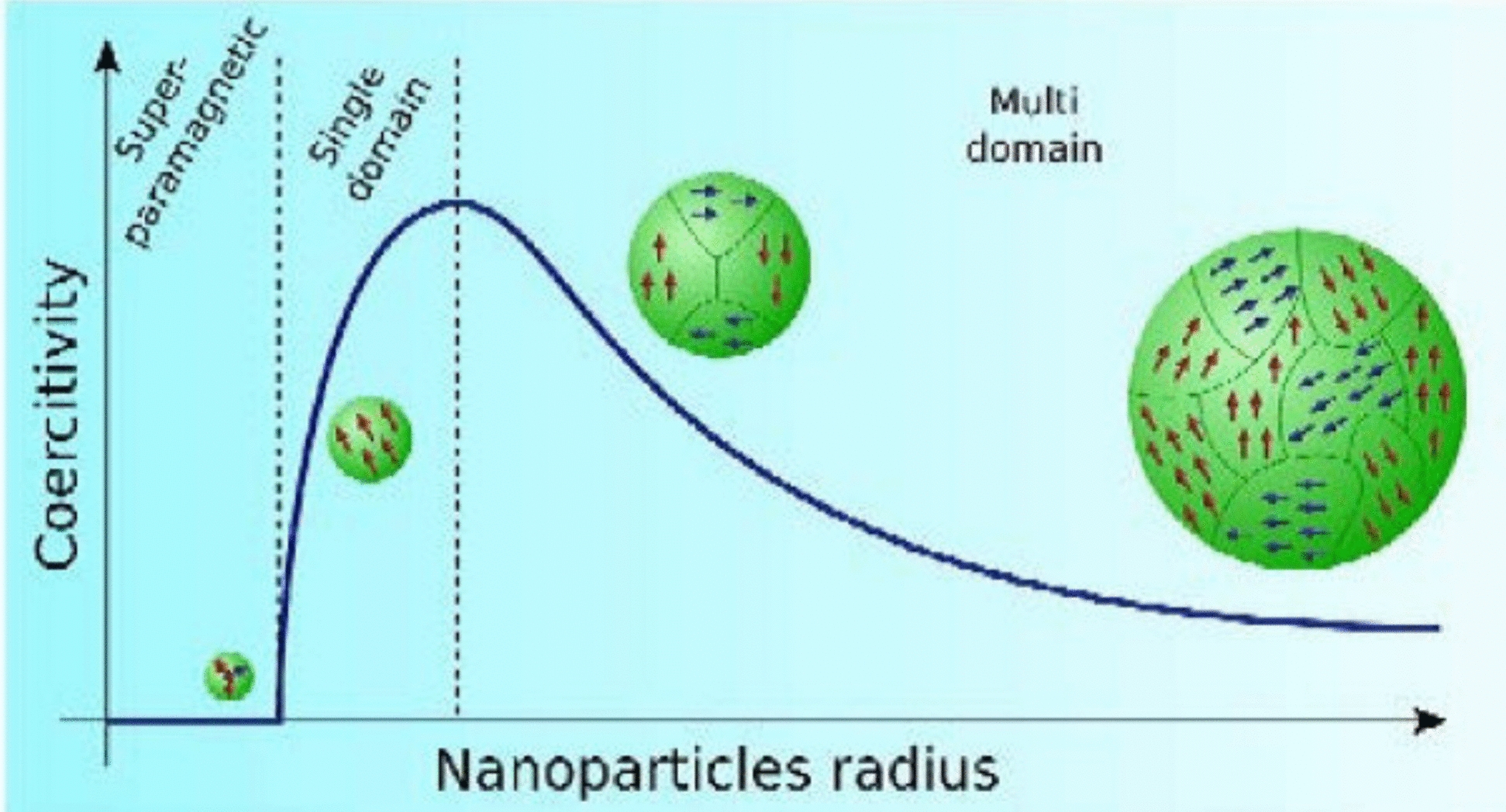
Schematic illustration of the coercivity–size relations of small particles [68]

One of the most important features of MNP is his superparamagnetic behavior. When particle sizes fall below a certain critical diameter—usually 20–30 nm—they show super paramagnetism [64]. This function eliminates the risk of particle aggregation due to remaining magnetism, especially to avoid obstacles in capillaries or cause long-term tissue accumulation [65]. Figure 3 shows the effect of particle size on the magnetic behavior of the NPs. The crystalline structure plays an important role in determining magnetic properties. High crystallinity ensures uniformity in magnetic reaction, which is important for frequent performance in applications, such as MRI contrast improvement or magnetic drug correction [66]. Magnetic saturation, power, and residue are the most important parameters describing the magnetic behavior of MNPs. When it comes to synthesis, parameters, such as temperature, pH, precursor initial concentration, and response time, can be good for controlling these structural properties. Techniques, such as coprecipitation, thermal decomposition, and hydrothermal synthesis, are widely used to achieve the desired structural and magnetic features [67].

### Biocompatibility and safety profiles

**Fig. 4:**
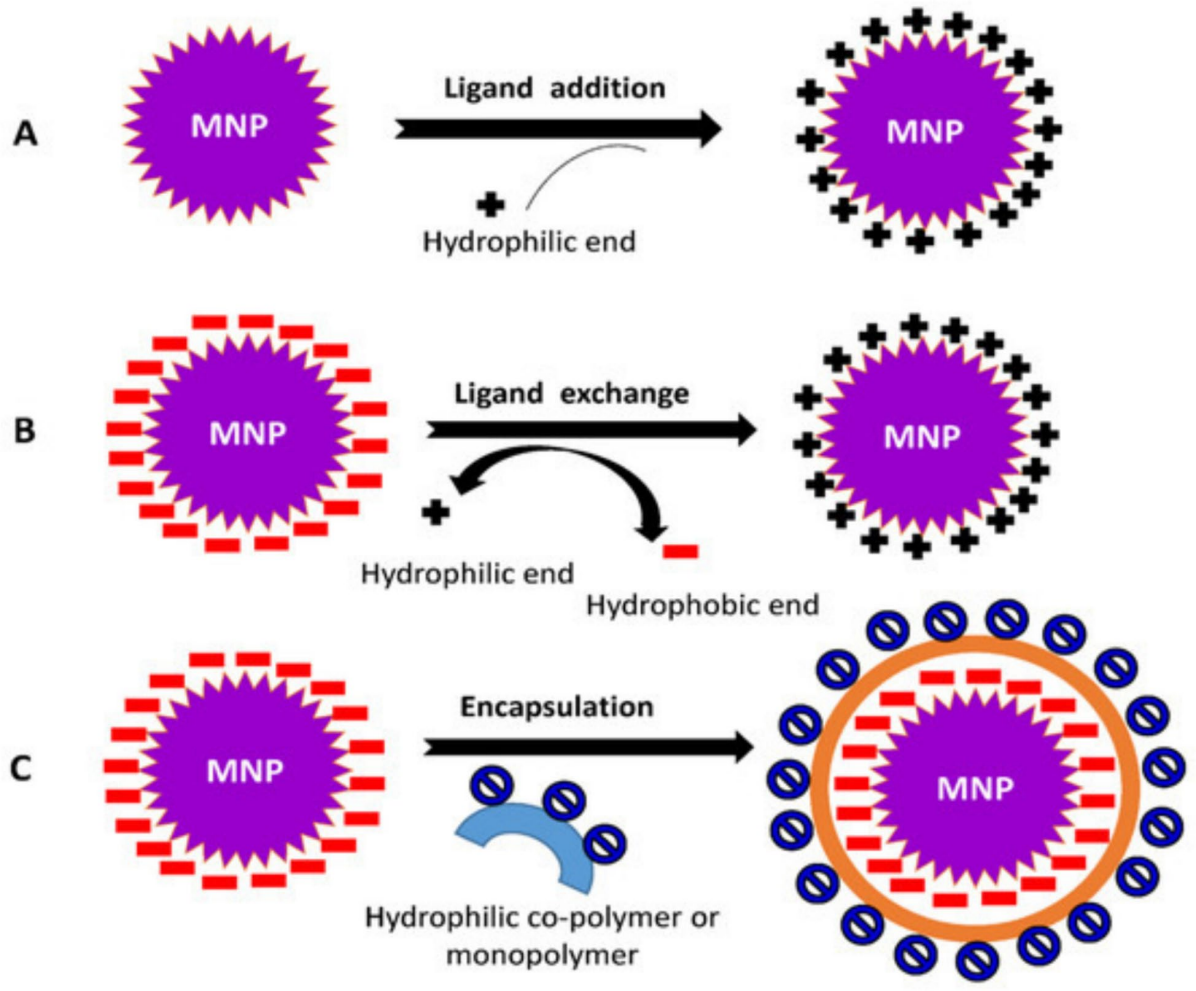
Surface modifications used to enhance MNP biocompatibility [75]

Biocompatibility is one of the most important criteria for clinical use of MNP. Since these particles intend to be used in biological systems, their interaction with cells, tissues, and organs should not induce side effects, such as inflammation, toxicity, or immune rejection [69]. The composition of MNP plays an important role in his compatibility [70]. Iron-oxide NPs (Fe_3_O_4_ or **γ**-Fe_2_O_3_) are generally recognized as safe (GRAS) by the FDA when produced under controlled conditions. They degrade to natural iron ions that can be included in the normal metabolic processes in the body [71]. However, surface modifications are necessary to prevent aggregation and increase the stability of the physical environment as indicated in Fig. 4.

Cytotoxicity is often evaluated through in vitro analyses using human and animal cell lines. These tests consider viability, oxidative stress, apoptosis, and membrane integrity after exposure to nanoparticles [72]. MNP synthesized through green methods has shown excellent performance in such tests, usually more than 90% of the cell's viability at high concentrations. This low toxicity is attributed to the lack of harmful chemicals and the lack of natural detergents used in the synthesis process [33].

One challenge in clinical translation is the long-term fate of nanoparticles. Biodegradation rates, excretion pathways, and possible interactions with other medications must be fully understood [73]. Studies using animal models have shown that surface-modified MNPs degrade over time and are gradually excreted without notable toxicity [74].

## Mechanisms of targeted drug delivery

Targeted drug delivery represents a major progress in oncology, aimed at increasing medical effect by reducing side effects. MNPs have proven to be effective carriers in this context because of their ability to locate in tumor tissue under the guidance of external magnetic fields. Targeted delivery can be classified into passive and active targeting strategies.

Passive targeting is based on the enhanced permeability and retention (EPR) effect observed in tumor vasculature. Tumor tissue contains blood vessels dripping and poor lymphatic drainage, which allows appropriate NPs (usually 10–200 nm), preferably at the tumor site. MNP can be constructed to take advantage of this natural phenomenon by adjusting the size and surface charge.

**Fig. 5:**
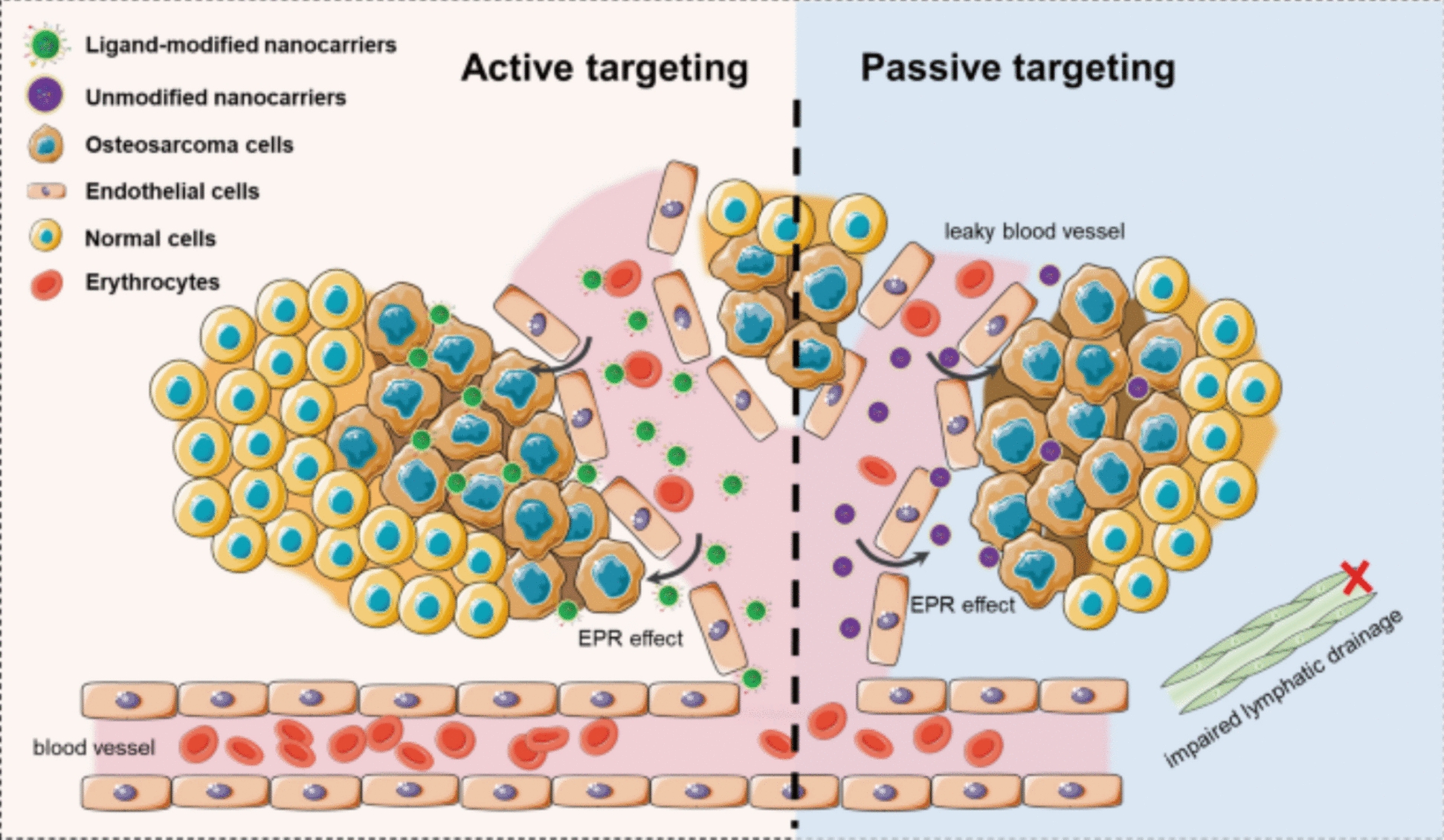
A schematic illustration of active targeting and passive targeting of nano-delivery system in anti-tumor therapy. Adapted from [76]

Active targeting involves functionalization of MNP surfaces with specific ligands, antibodies, peptides, or aptamers that recognize and bind molecular markers that are large over the cancer cells. General goals include folate receptors, HER2/NEU, and transferrin receptors. This selective bond increases cellular recording through the receptor-medieval endocytosis and improves drug accumulation at the target site. Figure 5 shows a schematic illustration of the two main ways to use magnetic nanoparticles to deliver drugs to specific areas: passive targeting and active targeting. Passive targeting takes advantage of the enhanced permeability and retention (EPR) effect that happens in tumors, which lets nanoparticles build up at the tumor site. Active targeting, on the other hand, uses surface changes like ligands or antibodies to bind specifically to cancer cell receptors, which makes the targeting more selective [76]. This figure shows how important both targeting mechanisms are for making cancer treatments more effective and less likely to cause side effects.

**Fig. 6:**
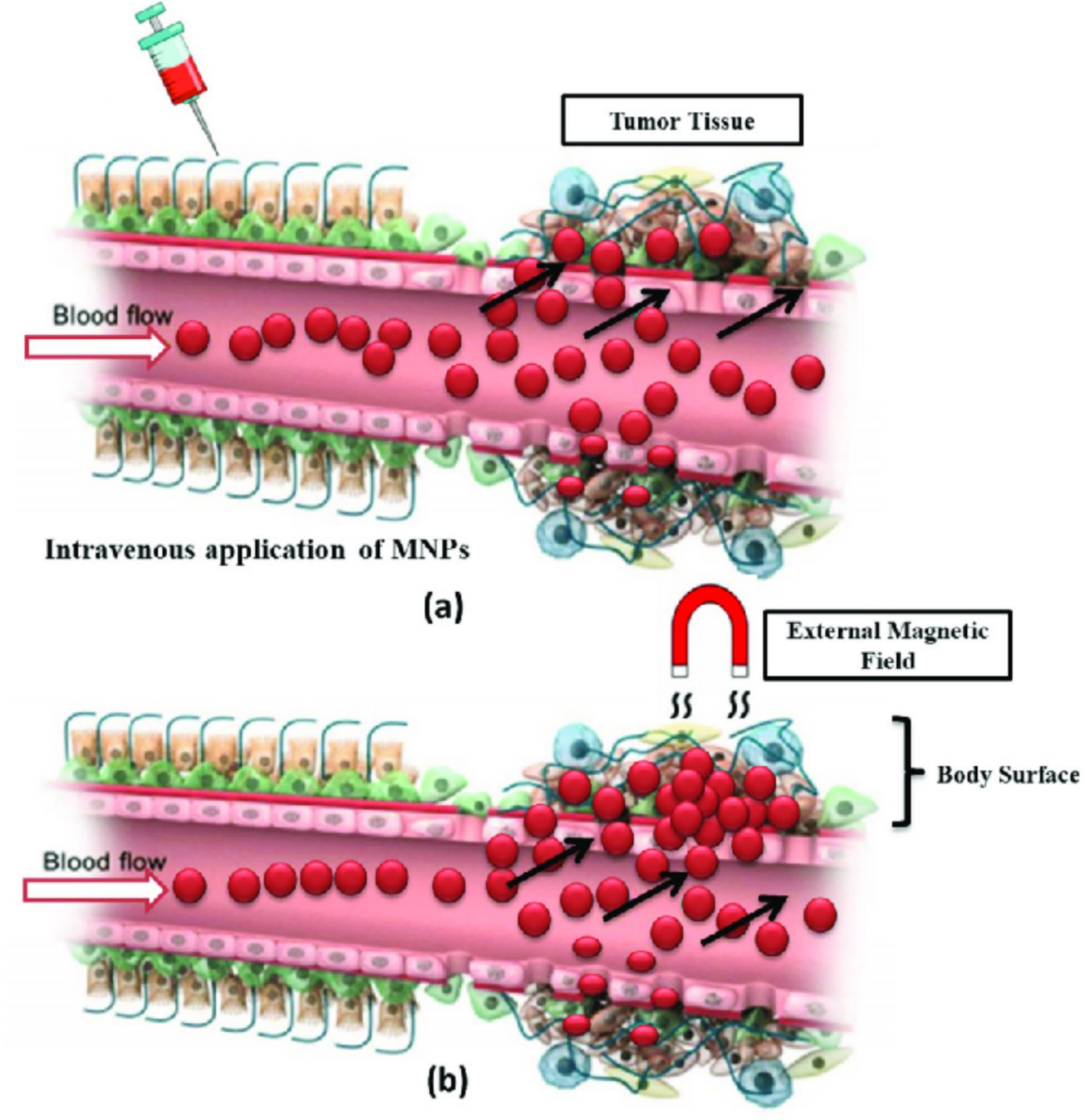
External magnetic field guiding MNPs to tumor site. Adapted from [77]

Figure 6 shows how an outside magnetic field can help magnetic nanoparticles find their way to tumor sites [77]. When a magnetic field is used, MNPs can be moved to a specific area. This makes it easier to deliver drugs to the tumor and lessens the risk of harming healthy tissues. This process makes MNPs work better as a treatment by letting more of the drug build up at the tumor site. This improves treatment results and lowers systemic toxicity. The figure shows how magnetic targeting could improve cancer treatment. This method increases the distribution of medical payload by reducing contact with healthy tissue. When biodegradable coatings and stimulation are connected with the mechanism of post-release (e.g., pH-sensitive polymer), MNPs may release medications specifically within tumor microenvironment. When the cancer cells are inner, MNPs usually leave its medical cargo either through the environmental trigger (e.g., acidic pH, enzyme) or through the decline of nanoparticles. This localized release increases intracellular drug concentrations and can start apoptosis or prevent proliferative pathways. Table 1 describes the targeting mechanisms of nanoparticles-based targeted delivery systems. Enhanced permeability and retention (EPR) effects are potentially beneficial; nevertheless, the targeted efficiency of magnetic nanoparticles (MNPs) may vary due to the heterogeneity of malignancies [78]. Tumor vasculature, genetic anomalies, and varying quantities of extracellular matrix (ECM) components can all make it harder for MNPs to accurately target certain tumor locations [79]. The diversity is made worse by the fact that it is hard to change the size and surface properties of MNPs to make sure that drugs are given out evenly across different forms of cancer [80].

**Table 1:** Key mechanisms of magnetic nanoparticle-based targeted delivery

Targeting mechanism	Description	Benefit
Passive targeting	EPR effect in tumors	Selective tumor accumulation
Active targeting	Ligand-receptor binding	Enhanced specificity
Magnetic targeting	External magnetic field	Controlled localization
pH-responsive release	Tumor microenvironment	Site-specific drug release
Hyperthermia	Magnetic heating	Synergistic therapeutic effect

### Concept of targeted therapy and mechanisms of action in cancer cells

Targeted therapy is a precision medicine approach that focuses on interfering with specific molecular targets involved in cancer growth and progression. Unlike traditional chemotherapy that affects all rapidly dividing cells, targeted therapy aims to selectively inhibit the activity of molecules—typically proteins or receptors—that are either overexpressed or mutated in cancer cells. This approach results in more effective treatment with fewer side effects.

A major tenet of targeted therapy is identifying cancer-specific biomarkers. These can be receptors, such as HER2, EGFR, VEGF, and oncogenic kinases, such as BCR-ABL or ALK. Targeted medications, which can be monoclonal antibodies or small molecule inhibitors, are specifically designed to bind to or inhibit these protein molecules. This binding prevents the cancer cell signaling cascades, prevents proliferation, induces apoptosis, and inhibits metastasis.

**Fig. 7:**
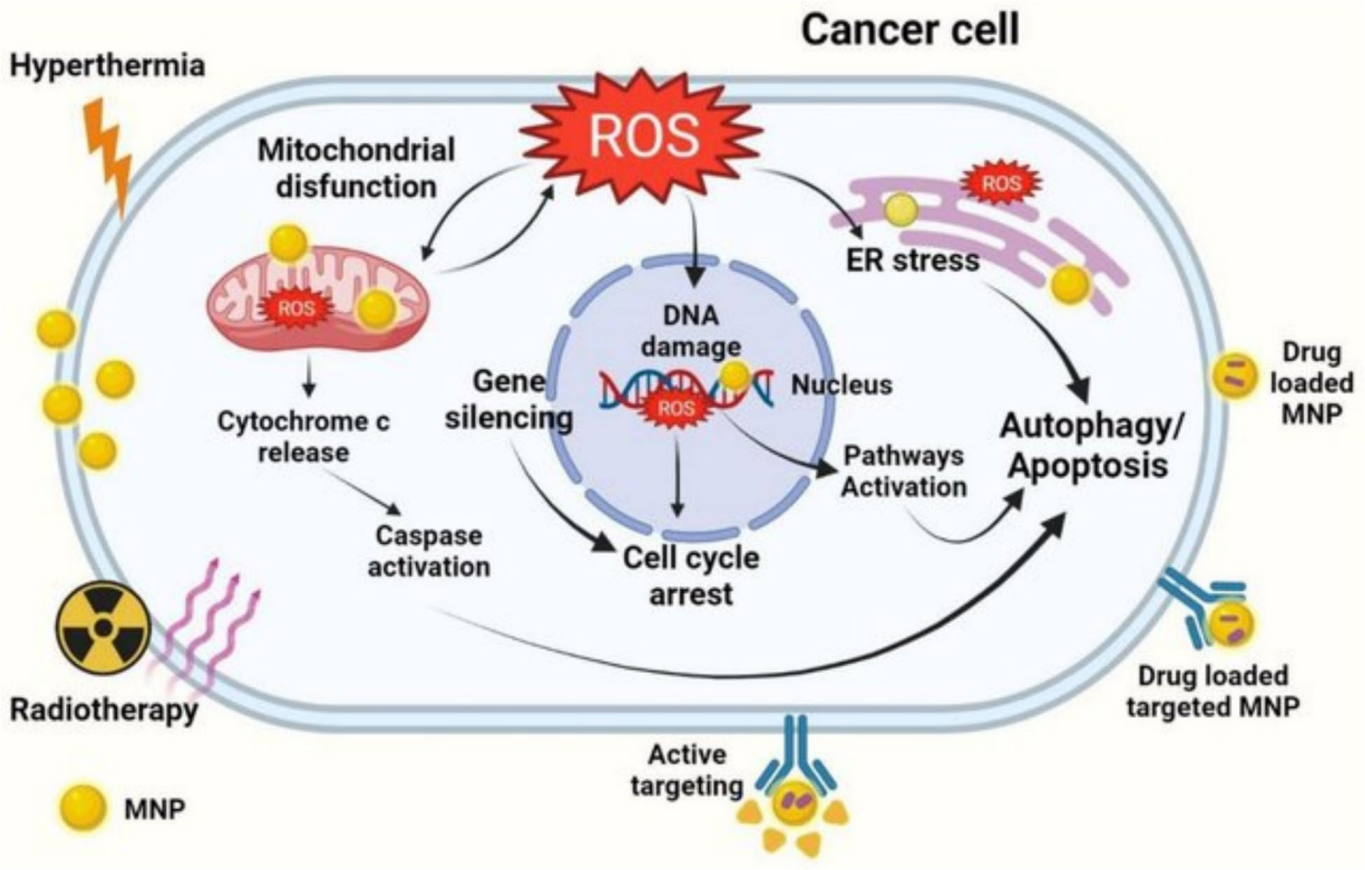
Mechanisms of anticancer effect of magnetic nanoparticles inside cancer cells. Adapted from [81]

To understand how therapeutic efficacy is accomplished in cancer by magnetic nanoparticles (MNPs), it is necessary to understand how to develop and utilize them. MNPs may utilize a variety of actions when acting as a drug delivery vehicles and/or active agent. Once a primary tumor cell engulfs MNP(s), they can induce release of cytotoxic agents, cause intracellular signaling disruption, induce oxidative stress, and induce programmed cell death (apoptosis). Figure 7 shows the different ways that magnetic nanoparticles can kill cancer cells. Once inside, MNPs can let out drugs in response to changes in the environment, like changes in pH or enzyme activity. MNPs can also make reactive oxygen species (ROS), which cause oxidative stress and damage DNA, which leads to apoptosis. The figure also shows that when MNPs are exposed to an alternating magnetic field, they can cause thermal damage through magnetic hyperthermia, which works with chemotherapy to make it more effective. This figure backs up the idea that MNPs work in many ways, which can be used to make cancer treatment more effective. The most prominent route of MNP’s internalization is with receptor-mediated endocytosis. Functionalized nanoparticles interact and bind with specific receptors on the cancer cell surface and will be engulfed into endosomes. The intracellular environment has variations in pH or enzyme activity to trigger drug release from the nanoparticle to elicit a local effect. The higher drug concentration within the cancer cell will have a greater cytotoxic effect while protecting body surrounding healthy tissue.

Furthermore, MNPs can also have therapeutic effects. Iron-oxide cores can produce reactive oxygen species (ROS) through Fenton-like reactions, which can cause oxidative stress and damage components of the cell, such as DNA, proteins, and lipids. Oxidative damage can induce mitochondrial dysfunction and loss of membrane potential, followed by activation of apoptotic pathways.

Another mechanism is thermal therapy. In the presence of an alternating magnetic field, MNPs can generate localized heat (magnetic hyperthermia), which increases the tumor tissue temperature up to a level that causes protein denaturation and ultimately cell death. Hyperthermia can also provide a synergistic effect on chemotherapy or radiation, thereby enhancing the therapeutic effect.

MNPs impart a multimodal mechanism of action in cancer cells, that is, delivery of drugs, apoptosis induction, cellular homeostasis disruption, and thermal ablation as an agent. These mechanisms can occur individually and/or in concert to improve efficiency and limit drug resistance.

### Case studies of magnetic nanoparticles in targeted therapy

Multiple preclinical and clinical studies have established the success of magnetic nanoparticles (MNPs) for site-specific delivery of chemotherapeutic agents to tumor sites. These case studies illustrate (1) the potential to obtain site-specific therapy, (2) reduced systemic exposure, and (3) enhanced therapeutic efficacy for MNP-based systems. Different types of drugs, such as doxorubicin, paclitaxel, and cisplatin, have been successful incorporated MNPs for targeted site-specific cancer therapy.

**Fig. 8:**
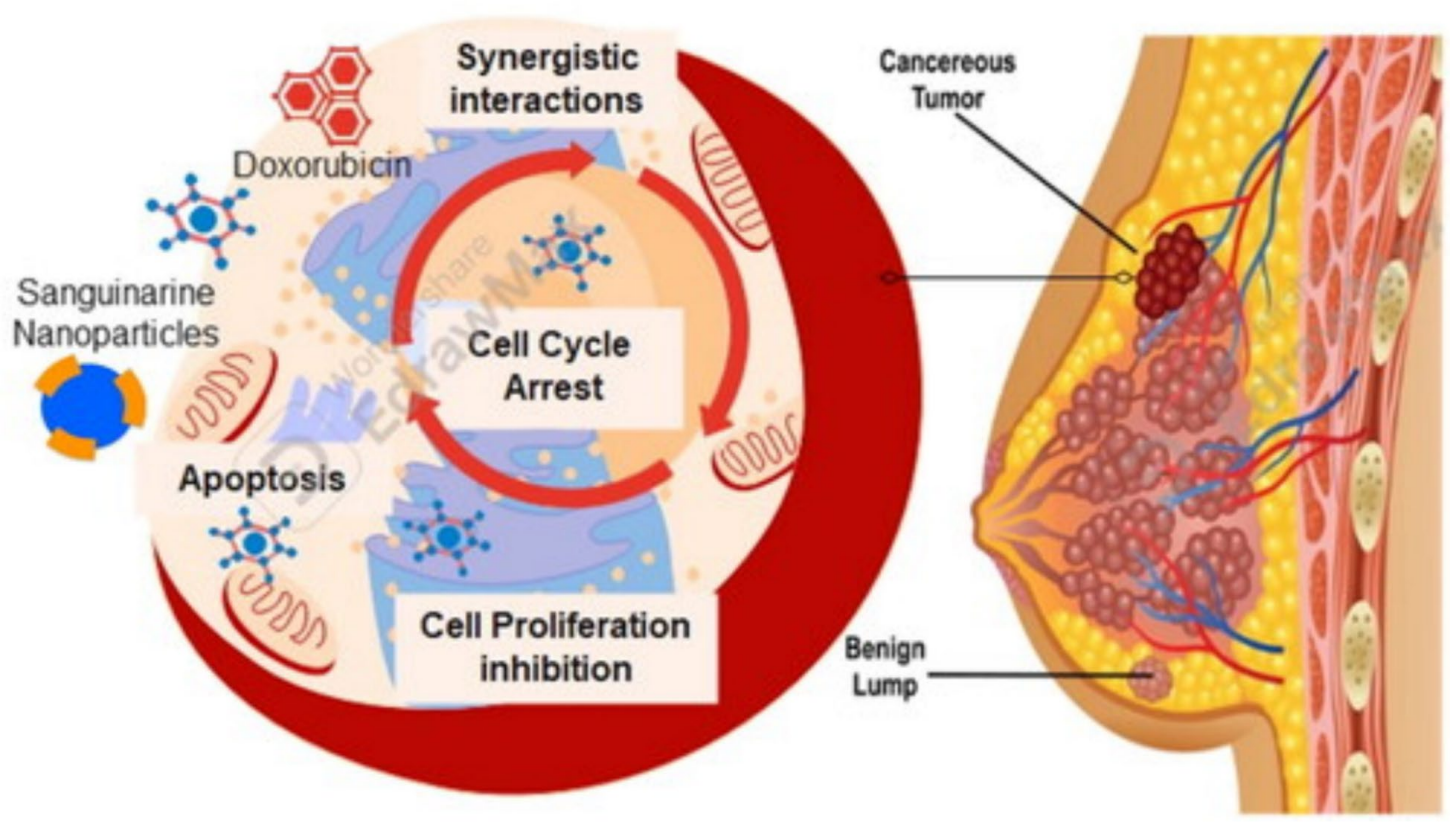
MNP-targeted delivery of doxorubicin to breast cancer cells. Adapted from [82]

As an example, doxorubicin-loaded MNPs were used in a murine bear cancer study as depicted in Fig. 8**.** Doxorubicin-loaded magnetic nanoparticles were designed with folic acid to preferentially target folate receptors that are overexpressed in cancer cells. The MNPs in this study exhibited an average particle size of 25 nm and a drug-loading concentration of 10 mg/mL. The MNPs retained the medication at the tumor site significantly more effectively when an external magnetic field was applied. This resulted in a 60% reduction in tumor volume relative to control groups. The quantity of doxorubicin in the MNP formulation was modified to ensure a gradual release over 48 h, with approximately 85% being dispensed over this timeframe. Histological analysis verified no cardiotoxicity, a significant disadvantage of unencapsulated doxorubicin [82].

In another study investigating paclitaxel-conjugated magnetic nanoparticles (MNPs) for the treatment of ovarian cancer, polyethylene glycol (PEG) was utilized to coat the MNPs. This prolonged their presence in the body and prevented premature excretion. The mean particle size of these MNPs was 30 nm, and they contained 15 mg/mL of paclitaxel. The MNPs containing paclitaxel were conjugated to antibodies that specifically target the CA-125 antigen, an indicator of ovarian cancer. When the medication was exposed to an external magnetic field, the accumulation at the tumor site significantly increased. Following a fortnight of treatment, the tumor volume was diminished by 75%. The rats administered paclitaxel-loaded MNPs had a markedly higher survival rate (90%) in contrast to the control group (50%). The pH-responsive polymer coating on the MNPs regulated the rate of paclitaxel release. The medication was administered over a period of 72 h and remained effective long after the treatment concluded [83]. These case studies demonstrate the potential of MNPs for targeted drug delivery, but the results also underscore the need for precise control over nanoparticle characteristics, including size, surface functionalization, and drug-loading concentration, to achieve optimal therapeutic outcomes. Further studies with larger sample sizes and varied tumor types are needed to validate the reproducibility and generalizability of these findings.

### Comparative studies with traditional drug carriers

MNPs have proven to be adaptable, heterogeneous drug delivery vectors that can cross multiple barriers for traditional drug carriers [84]. However, unanswered questions regarding limited targeting ability, premature drug release, lack of stability in circulation, and imaging capabilities completely abolish its possible use to any other systems [25, 85].

Many comparative studies have tested MNP and compared them side-by-side to traditional carriers [86–88]. Table 2 lists the comparative features of MNPs-Based drug delivery systems and traditional drug delivery systems.

**Table 2:** Comparative features of MNPs- based- drug delivery systems and traditional drug delivery systems

Property	Traditional drug delivery systems	MNPs-based drug delivery systems
Solubility	Limited solubility in aqueous systems	Increase the drug solubility through surface modifications (e.g., PEGylation)
Shape	Often spherical, liposomal, or polymeric	Typically spherical, but can be tailored for specific applications
Size	Varies (typically larger particles)	Small size (typically 10–200 nm); can be precisely controlled
Surface functionalization	Requires additional functionalization for targeting (e.g., antibodies, PEG)	Surface can be easily functionalized with ligands, antibodies, or polymers for active targeting
Magnetic targeting	No magnetic targeting capability	Magnetic targeting through external fields for precise tumor site localization
Biodistribution	Poor distribution within the body	Effective targeted delivery
Selectivity	Lack of selectivity	Reducing the amount of drug needed
Required dose	High dose required	Low dose required
Circulation time	Short circulation time; rapid clearance	Long circulation time due to surface modifications
Scalability	Well-established production methods, but still costly for large-scale use	Scalable but dependent on synthesis method; green synthesis is eco-friendly but still under development for large-scale production
Side effect	Higher side effect due to nonspecific distribution	Lower side effect due to targeted delivery and reduced off-target accumulation

The drug release properties are more personalized due to its stimuli-responsive coatings in MNP that are triggered by pH, temperature, or enzymes [88]. These coatings confirm that the material is released in the tumor microenvironment, while traditional carriers would have the potential for leaks and off-target effects [89].

Another advantage of MNPs lies in their imaging capability [90]. Traditional carriers lack intrinsic imaging properties and often require separate agents for diagnostics [91]. MNPs, particularly iron-oxide nanoparticles, provide contrast for magnetic resonance imaging (MRI), enabling simultaneous therapy and monitoring—known as theranostics [92].

## Challenges and limitations

Despite the considerable potential of magnetic nanoparticles (MNPs) in cancer therapy, several challenges and limitations must be addressed before they can be widely adopted in clinical practice. These limitations span across synthesis, scalability, reproducibility, regulatory compliance, and long-term safety [93].

One major challenge is the reproducibility of synthesis. The physicochemical properties of MNPs, including size, shape, surface charge, and magnetic behavior, are highly sensitive to synthesis conditions. Minor variations in temperature, pH, or precursor concentration can lead to significant inconsistencies. Ensuring batch-to-batch uniformity is critical for reproducibility and regulatory approval [94].

Another concern is particle aggregation. MNPs tend to aggregate in physiological environments due to van der Waals and magnetic dipole interactions [95]. Aggregation not only alters biodistribution but also reduces therapeutic efficiency and can cause embolism or unwanted accumulation in non-target organs [96]. Surface coatings, such as PEG and dextran, help improve stability but add complexity to the formulation [97].

Toxicity and clearance remain partially unresolved. While MNPs are often considered biocompatible, their long-term accumulation and degradation products (e.g., free iron ions) may induce oxidative stress or interfere with normal cellular processes. Comprehensive toxicological evaluations are necessary to ensure safety over extended periods, especially for repeated dosing regimens [69, 98].

Cost and scalability are also limiting factors. Green synthesis methods, though eco-friendly, are not yet optimized for industrial-scale production. High production costs and the need for advanced facilities may hinder widespread application in low-resource settings [99].

Drug release efficiency varies significantly depending on factors, such as the type of coating, particle size, and the external stimuli used to trigger drug release. MNPs with polymer or lipid-based coatings often show controlled release, but the release rate can be influenced by factors like the hydrophobicity of the drug and the stability of the coating under physiological conditions. Additionally, the inconsistency in release rates due to pH, temperature, and enzyme variations in the tumor microenvironment further complicates the reliability of MNPs as drug carriers [100].

Instability in Blood Circulation remains a significant issue. Upon intravenous administration, MNPs are subject to aggregation due to van der Waals forces and magnetic interactions. This aggregation leads to inconsistent biodistribution and affects their ability to effectively target tumor sites. The presence of proteins in the blood can cause nonspecific binding to nanoparticles, reducing their stability and effectiveness. To mitigate this, surface modifications such as PEGylation or the use of stabilizing agents like dextran have been employed, but these solutions are not universally effective [101].

Moreover, inconsistent targeting performance is another challenge for MNPs in clinical applications. Despite the theoretical advantages of the enhanced permeability and retention (EPR) effect, tumor heterogeneity can significantly impact nanoparticle accumulation and drug delivery efficiency. Variations in tumor vascularization and the microenvironment can hinder the precise targeting of MNPs, leading to suboptimal therapeutic outcomes. Active targeting strategies, such as ligand–receptor interactions, show promise in increasing selectivity, but challenges persist in achieving consistent targeting across different cancer types [102].

## Future perspectives

The future of magnetic nanoparticles (MNPs) in cancer therapy holds immense potential, particularly as research advances toward smarter, multifunctional, and personalized drug delivery systems. New innovations have the potential to address existing limitations, enhance treatment efficacy, and become compatible with emerging medical technologies. One avenue is the generation of stimuli-responsive MNPs that release drugs upon exposure to multiple environmental stimuli, e.g., changes in pH, temperature, magnetic fields, or enzymatic activity. These smart, on-demand delivery systems can allow for enhanced delivery control, resulting in precision and dynamic treatment regimens personalized to each tumor [103].

Another pathway of exploration involves the pairing of MNPs to other treatment methods. Combinations of MNPs coupled with immunotherapy, gene therapy, or radiotherapy will yield synergistic effects by attacking cancer along different mechanisms. These combinations could also potentially circumvent drug resistance and tumor heterogeneity challenges [104]. The field of personalized nanomedicine is rising where MNPs are designed and synthesized based on patient specific biomarkers and tumor profiles. This targeted approach will maximize delivery and minimize side effects [105, 106]. Additionally, artificial intelligence (AI) and machine learning also have a potential role in nanoparticle design, prediction, and optimization of nanoparticle formulations for maximal therapeutic outcomes [107]. MNPs are also exploring methods of real-time monitoring and feedback. Theranostic platforms may offer concurrent therapy and imaging for real-time monitoring, as well as the ability to change treatment paradigms [108].

## Conclusion

Magnetic nanoparticles made using green synthesis showed great potential to enhance cancer therapy. These unique multifunctional properties, with magnetic responsiveness, biocompatibility, and surface functionalization, provided support for drug delivery, hyperthermia, and theranostics. This review provides a comprehensive overview of the current research landscape of the green synthesis, characterization, and applications of MNP in drug delivery. While there has been a lot of positive progress, there still remain challenges related to large-scale fabrication, stability, controlled drug release, and regulatory approval. Innovations in design, combined with interdisciplinary integration in biotechnology, material science, and medicine, however, lead a promising path. Finally, MNPs are poised to become a critical component of personalized and precision medicine, enabling safer, more effective, and smarter cancer treatments in the near future.

## Data Availability

No datasets were generated or analysed during the current study.
